# Point-of-care testing for emergency assessment of coagulation in patients treated with direct oral anticoagulants including edoxaban

**DOI:** 10.1186/s42466-021-00105-4

**Published:** 2021-03-01

**Authors:** Florian Härtig, Ingvild Birschmann, Andreas Peter, Sebastian Hörber, Matthias Ebner, Matthias Sonnleitner, Charlotte Spencer, Paula Bombach, Maria-Ioanna Stefanou, Johannes Tünnerhoff, Annerose Mengel, Joachim Kuhn, Ulf Ziemann, Sven Poli

**Affiliations:** 1 Department of Neurology & Stroke, University Hospital, Eberhard-Karls University Tübingen, Hoppe-Seyler-Str. 3, 72076 Tübingen, Germany; 2 Hertie Institute for Clinical Brain Research, Eberhard-Karls University Tübingen, Tübingen, Germany; 3 Institute for Laboratory and Transfusion Medicine, Heart and Diabetes Center, Ruhr University, Bad Oeynhausen, Germany; 4 Department of Diagnostic Laboratory Medicine, Institue for Clinical Chemistry and Pathobiochemistry, Eberhard-Karls University Tübingen, Tübingen, Germany; 5 Department of Nephrology and Medical Intensive Care, Charité University Medicine, Berlin, Germany

**Keywords:** Point-of-care testing, POCT, Direct oral anticoagulants, DOAC, Non-vitamin K antagonist oral anticoagulants, NOAC, Anticoagulation reversal, Thrombolysis, Stroke

## Abstract

**Background:**

Direct oral anticoagulants (DOAC) including edoxaban are increasingly used for stroke prevention in atrial fibrillation. Despite treatment, annual stroke rate in these patients remains 1–2%. Rapid assessment of coagulation would be useful to guide thrombolysis or reversal therapy in this growing population of DOAC/edoxaban-treated stroke patients. Employing the Hemochron™ Signature Elite point-of-care test system (HC-POCT), clinically relevant plasma concentrations of dabigatran and rivaroxaban can be excluded in a blood sample. However, no data exists on the effect of edoxaban on HC-POCT results.

We evaluated whether edoxaban plasma concentrations above the current treatment thresholds for thrombolysis or anticoagulation reversal (i.e., 30 and 50 ng/mL) can be ruled out with the HC-POCT.

**Methods:**

We prospectively studied patients receiving a first dose of edoxaban. Six blood samples were collected from each patient: before, 0.5, 1, 2, 8, and 24 h after drug intake. HC-POCT-based INR (HC-INR), activated clotting time (HC-ACT+ and HC-ACT-LR), activated partial thromboplastin time (HC-aPTT), and mass spectrometry for edoxaban plasma concentrations were performed at each time-point. We calculated correlations, receiver operating characteristics (ROC) and test-specific cut-offs for ruling out edoxaban concentrations > 30 and > 50 ng/mL in a blood sample.

**Results:**

One hundred twenty blood samples from 20 edoxaban-treated patients were analyzed. Edoxaban plasma concentrations ranged from 0 to 512 ng/mL. HC-INR/HC-ACT+/HC-ACT-LR/HC-aPTT ranged from 0.7–8.3/78–310 s/65–215 s/19–93 s, and Pearson’s correlation coefficients showed moderate to very strong correlations with edoxaban concentrations (*r* = 0.95/0.79/0.70/0.60). With areas under the ROC curve of 0.997 (95% confidence interval: 0.991–0.971) and 0.989 (0.975–1.000), HC-INR most reliably ruled out edoxaban concentrations > 30 and > 50 ng/mL, respectively, and HC-INR results ≤1.5 and ≤ 2.1 provided specificity/sensitivity of 98.6% (91.2–99.9)/98.0% (88.0–99.9) and 96.8% (88.0–99.4)/96.5% (86.8–99.4).

**Conclusions:**

Our study represents the first systematic evaluation of the HC-POCT in edoxaban-treated patients. Applying sufficiently low assay-specific cut-offs, the HC-POCT may not only be used to reliably rule out dabigatran and rivaroxaban, but also very low edoxaban concentrations in a blood sample. Because the assay-specific cut-offs were retrospectively defined, further investigation is warranted.

**Trial registration:**

ClinicalTrials.gov, registration number: NCT02825394, registered on: 07/07/2016, URL

**Supplementary Information:**

The online version contains supplementary material available at 10.1186/s42466-021-00105-4.

## Background

Edoxaban, alongside other direct oral anticoagulants (DOAC), is increasingly replacing vitamin K antagonists (VKA) for the treatment and prevention of venous and arterial thromboembolism including ischemic stroke [1]. Similar to the other DOAC, edoxaban has gained approval for these indications by providing comparable efficacy and improved safety. Nonetheless, annual stroke risk in edoxaban-treated patients with atrial fibrillation remains just above 1% [2], major bleeding events may occur and hemostatic management in emergency surgery is complicated. In order to make an informed decision on whether to apply or withhold thrombolysis in case of ischemic stroke or reverse the anticoagulant effect prior to surgical interventions by administration of an expensive and potentially prothrombotic antidote [3] or coagulation factors [4], the coagulation status of the patient must be known. Calibrated anti-Xa activity assays are recommended by guidelines as state-of-the-art for coagulation assessment during edoxaban therapy [5, 6]. Unfortunately, these assays are not available on any commercial point-of-care test system (POCT), and laboratory-based coagulation testing clearly limits emergency decision making due to their long turn-around-times [7].

In analogy to our previous research conducted with apixaban, dabigatran, and rivaroxaban [8–10], we aimed to determine whether available point-of-care coagulation assays also allow the exclusion of very low but clinically relevant edoxaban levels in a blood sample [11]. We hypothesized that edoxaban plasma concentrations above 30 and 50 ng/mL (i.e. the two current guideline thresholds for thrombolysis in acute ischemic stroke [5, 12], urgent surgical procedures [13], and anticoagulation reversal in intracranial hemorrhage [5] or other serious bleeding [13]) can be ruled out in a blood sample with help of the Hemochron™ Signature Elite POCT (HC-POCT; Werfen, Barcelona, Spain) and its test cartridges for measuring the prothrombin time/INR (HC-INR), activated clotting time (HC-ACT+ and HC-ACT-LR), or activated partial thromboplastin time (HC-aPTT).

## Methods

### Study design, setting and eligibility criteria

Single-center, prospective diagnostic trial with blinded outcome assessment, registered on ClinicalTrials.gov under NCT02825394. The study was conducted at the Department of Neurology & Stroke of Tübingen University Hospital, a tertiary care facility. We planned to enroll 20 patients after ischemic stroke receiving their first dose of edoxaban for secondary prevention of thromboembolism. Patients with abnormal coagulation values at baseline (Quick < 70% or aPTT > 40 s), history of coagulopathy or subjects who had received VKA or DOAC within 14 days, low-molecular-weight heparins within 24 h, or unfractionated heparin within 12 h before first DOAC intake were excluded to rule out interference with measurements. Use of anti-platelet drugs was permitted.

### Sample collection

Six blood samples were collected from each subject via a venous catheter or by direct venipuncture before first intake of edoxaban, 0.5, 1, 2, 8, and 24 h after intake. This was done in order to cover a wide range of edoxaban plasma concentrations including a high number of samples with low concentrations around the above-mentioned 30 and 50 ng/mL treatment thresholds.

### Coagulation testing

Whole blood was drawn directly into a syringe (Injekt, BBraun, Melsungen, Germany) and used to conduct HC-INR, HC-ACT+, HC-ACT-LR, and HC-aPTT on a HC-POCT within 15 s of sampling. Additional blood was drawn into a standard blood sampling tube for coagulation assays (S-Monovette Citrate 3.2%, Sarstedt, Nümbrecht, Germany) and sent to the central laboratory of Tübingen University Hospital for laboratory-based calibrated anti-Xa activity, using the Innovance Heparin assay on a Sysmex CS-5100 (both Siemens Healthineers, Erlangen, Germany). Additionally, at baseline, a full blood count, baseline coagulation tests, inflammatory markers, protein/albumin as well as liver and kidney function tests were performed. Further samples of citrated whole blood were centrifuged at 2500 g for 15 min to yield citrated plasma and stored at our center at − 80 °C; one sample per time point was later shipped to the Institute for Laboratory and Transfusion Medicine at the Heart and Diabetes Center of Ruhr University (Bad Oeynhausen, Germany) for ultra-performance liquid chromatography–tandem mass spectrometry (UPLC-MS/MS), which was performed in a manner previously described [14] as a gold-standard method to determine exact edoxaban plasma concentrations. All POCT and laboratory-based tests were performed according to manufacturers’ instructions by thoroughly trained investigators and technicians.

### Blinding

All POCT operators were blinded to results of UPLC-MS/MS and laboratory-based coagulation assays. External technicians conducting UPLC-MS/MS were blinded to results of POCT and laboratory-based coagulation assays, as well as patient number and sampling time point. Fully automated laboratory-based measurements were conducted during routine operation at our central laboratory where technicians were blinded to POCT results and UPLC-MS/MS.

### Statistical analyses

Pearson’s correlation coefficient was used to estimate the strength of correlation between POCT results and actual edoxaban plasma concentrations determined by UPLC-MS/MS. Evans’ suggestions were used to describe the strength of correlation [15].

When assessing diagnostic accuracy of POCT, assay-specific cut-offs were evaluated regarding their capability to categorize blood samples according to the two edoxaban concentration thresholds, i.e., 30 and 50 ng/mL. Specificity was defined as the percentage of samples containing edoxaban concentrations > 30/50 ng/mL that were correctly identified as such by elevated POCT results, and thus, as theoretically belonging to a patient who should not receive thrombolysis, or would require reversal therapy in case of life-threatening hemorrhage or prior to surgery. Correspondingly, sensitivity was defined as the percentage of samples containing edoxaban plasma concentrations ≤30/50 ng/mL that were correctly identified as such by low POCT results. Positive predictive value was defined as the percentage of samples with edoxaban plasma concentrations ≤30/50 ng/mL of all samples identified as such by low POCT results, and negative predictive value was defined as the percentage of samples with edoxaban concentrations > 30/50 ng/mL of all samples identified as such by elevated POCT results. Likelihood (sensitivity/1-specificity) indicates how much more likely a low POCT result is found in samples containing ≤30/50 ng/mL compared to samples containing > 30/50 ng/mL of edoxaban. In analogy to other authors [16], a misprediction percentage (1-specificity) is additionally provided, which represents how often edoxaban plasma concentrations > 30/50 ng/mL occurred despite POCT results that indicate concentrations ≤30/50 ng/mL.

For both thresholds and each POCT assay, receiver operating characteristics (ROC) curves were drawn and the area under the ROC curve (AUROC) was calculated. An ‘ideal’ assay-specific cut-off for POCT results was defined by the lowest possible value that yielded a specificity of ≥95% (misprediction percentage ≤ 5%) in order to avoid false negative results, which would constitute a significant safety issue.

95% confidence intervals (95%-CI) for proportions (specificity, sensitivity, positive and negative predictive values) and likelihood ratios were calculated according to the efficient-score method as described by Newcombe [17] using the free online VassarStats Clinical Calculator 1 (*VassarStats Clinical Calculator 1*) [18]. SPSS version 24 (IBM, Armonk, NY, USA) was used for all other statistical analyses.

## Results

### Patient population

Between October 2016 and May 2017, twenty patients receiving a first dose of edoxaban for secondary stroke prevention were included in the study (see Table 1 for a summary of patients’ baseline characteristics). In all cases, six blood samples were obtained, leading to 120/119/117/117 measurements of HC-INR/HC-ACT+/HC-ACT-LR/HC-aPTT, 119 measurements of laboratory-based anti-Xa activity, and 120 UPLC-MS/MS measurements of edoxaban plasma concentration. Few measurements could not be obtained for technical reasons.

**Table 1:** Patients’ baseline characteristics (*N* = 20)

Age	66 ± 10.5 years
Female sex	8 (40%)
Edoxaban dose	60 mg daily: 15 (75%) 30 mg daily: 5 (25%)
Body weight	80.5 ± 20.1 kg
Body Mass Index (BMI)	27.0 ± 5.9 kg/m^2^
Glomerular filtration rate (Cockcroft-Gault)	83 ± 26 mL/min/1.73m^2^
*Risk factors*	
Arterial hypertension	16 (80%)
Hyperlipidemia	7 (35%)
Diabetes mellitus	1 (5%)
History of stroke	20 (100%)
Congestive heart failure	2 (10%)
Coronary heart disease	7 (35%)
History of myocardial infarction	5 (25%)
Smoking	4 (20%)
*Indication for edoxaban therapy*	
Atrial fibrillation	12 (60%)
Stroke associated with patent foramen ovale	6 (30%)
Embolic stroke of undetermined source	2 (10%)
*Concomitant antiplatelet therapy*	4 (20%)

Continuous variables are displayed as mean ± standard deviation. Nominal variables are displayed as absolute quantity (percentage)

### Correlation between laboratory-based calibrated anti-Xa activity or HC-POCT and edoxaban concentrations

**Fig. 1:**
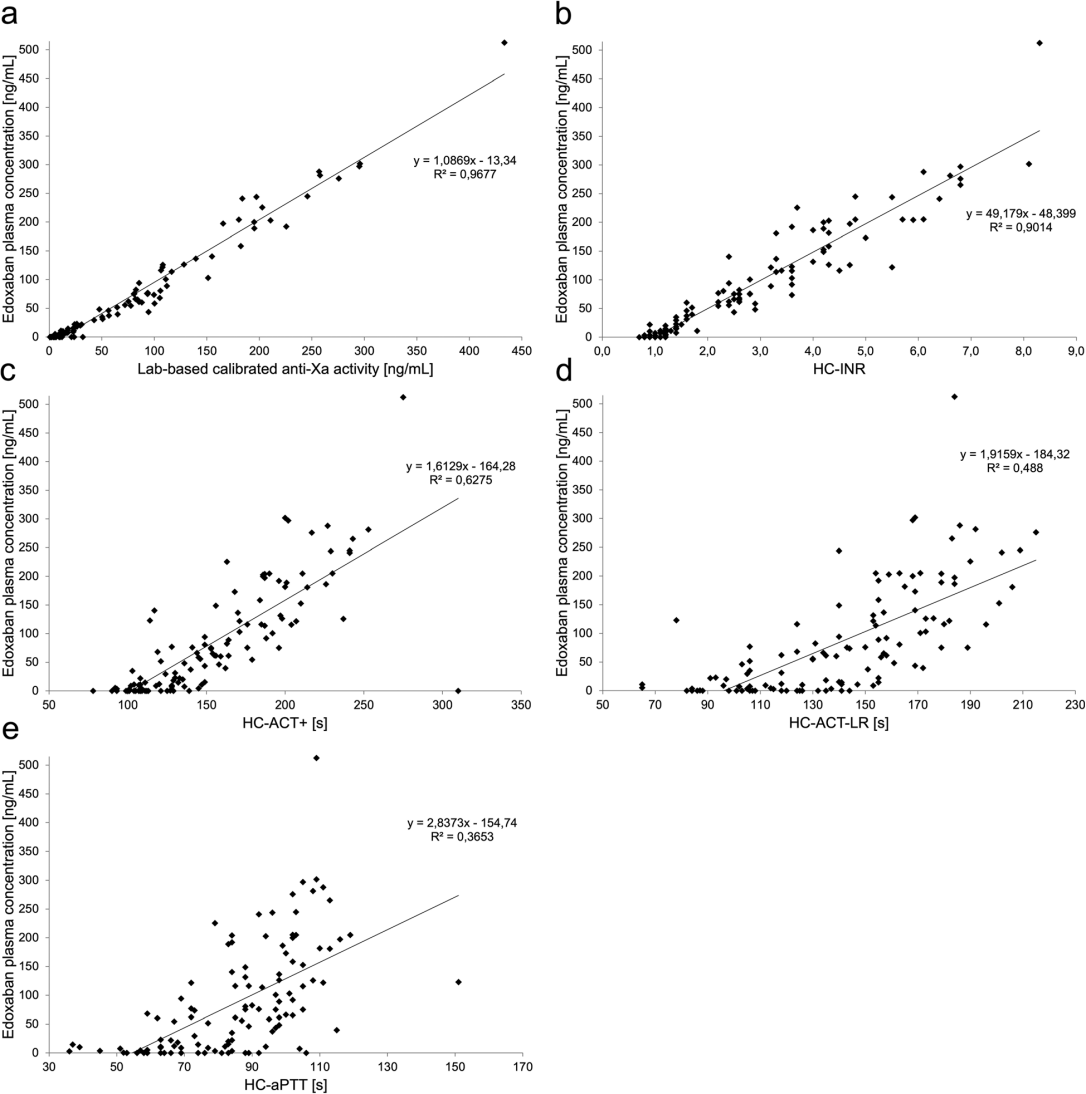
Scatter plots visualizing the correlation of (**a**) laboratory-based calibrated anti-Xa activity and (**b** to **e**) the results of all four Hemochron™ Signature Elite point-of-care test system-based coagulation assays with edoxaban plasma concentrations determined by mass spectrometry. HC-INR, HC-ACT+, HC-ACT-LR, HC-aPTT = Hemochron™ Signature Elite point-of-care test system-based international normalized ratio, activated clotting time, and activated partial thromboplastin time

Edoxaban plasma concentrations as measured by UPLC-MS/MS ranged from 0 to 512 ng/mL, whilst 50 (42%) samples contained ≤30 ng/mL and 57 (47.5%) samples contained ≤50 ng/mL. Laboratory-based calibrated anti-Xa activity ranged from 0 to 433 ng/mL, and HC-INR/HC-ACT+/HC-ACT-LR/HC-aPTT from 0.7–8.3/78–310 s/65–215 s/19–93 s. Correlation between laboratory-based calibrated anti-Xa activity and actual edoxaban plasma concentrations was very strong (*r* = 0.98; *p* < 0.001). Very strong correlation was also found for HC-INR and edoxaban plasma concentrations (*r* = 0.95; p < 0.001). Strong correlation was found for HC-ACT+ (*r* = 0.79; *p* < 0.001) and HC-ACT-LR (*r* = 0.70; *p* < 0.001), and a moderate correlation for HC-aPTT (*r* = 0.60; *p* < 0.001) (see Fig. 1).

### Diagnostic accuracy of laboratory-based calibrated anti-Xa activity and HC-POCT to detect low edoxaban concentrations around current treatment thresholds

**Fig. 2:**
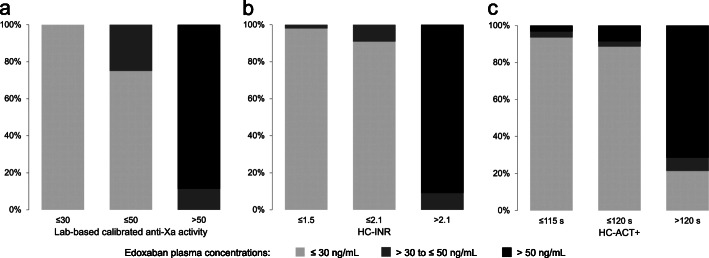
Receiver operating characteristics curves found for laboratory-based calibrated anti-Xa activity all four Hemochron™ Signature Elite point-of-care test system-based coagulation assays when testing for ruling out samples containing > 30 ng/mL (solid line) and > 50 ng/mL (dashed line)

**Fig. 3:**
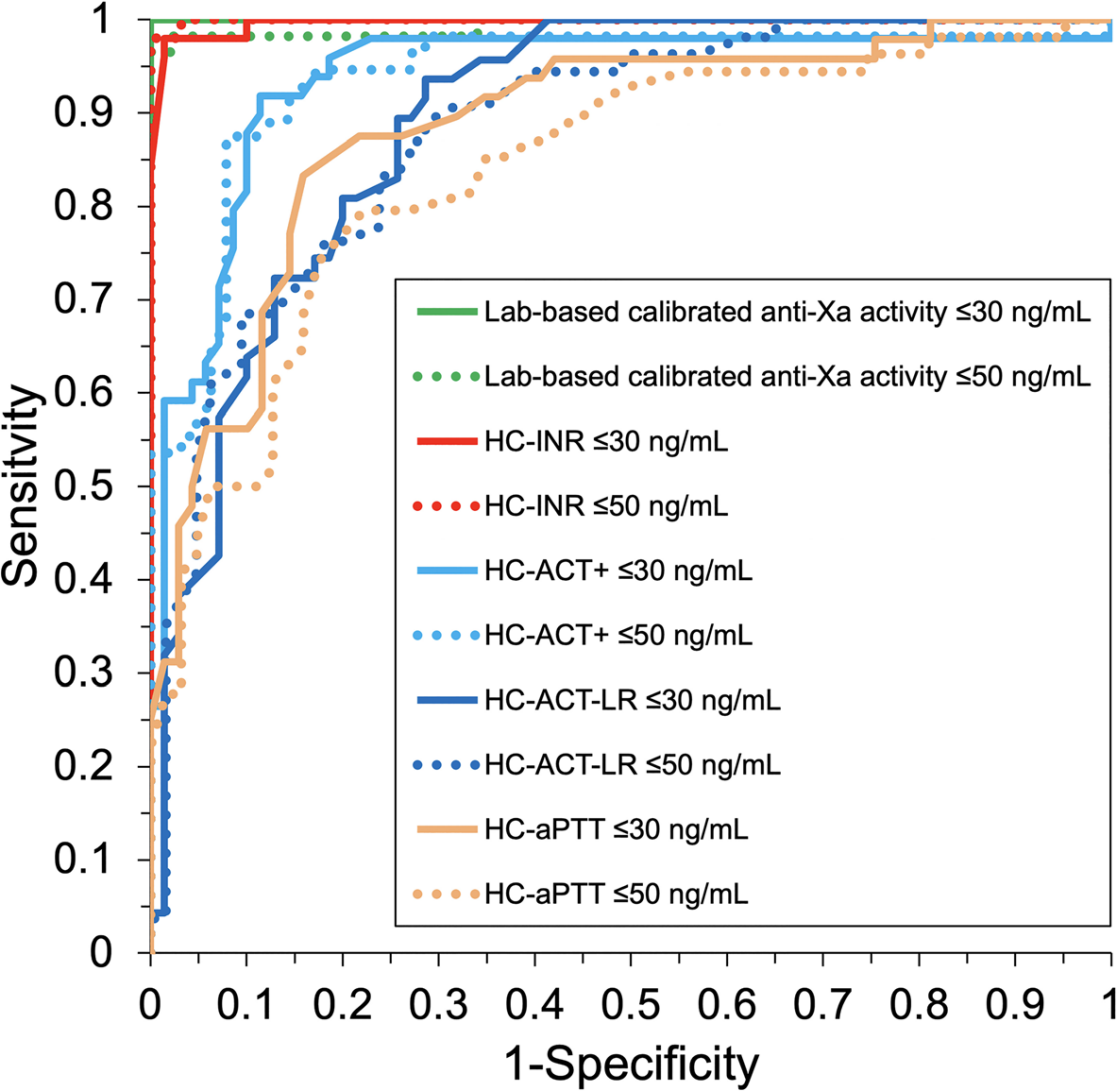
Percentage of edoxaban concentrations up to 30 ng/mL (light gray), > 30 and ≤ 50 ng/mL (dark gray) and > 50 ng/mL (black) found at corresponding laboratory-based calibrated anti-Xa activity and the ‘ideal cut-offs’ for Hemochron™ Signature Elite point-of-care test system-based prothrombin time/INR (HC-INR) and activated clotting time (HC-ACT+)

Using the thresholds of ≤30 and ≤ 50 ng/mL, laboratory-based calibrated anti-Xa activity reached the highest AUROC of 1.000 (95%-CI: 1.000–1.000) and 0.994 (95%-CI: 0.981–1.000), respectively, followed by HC-INR with AUROC of 0.997 (95%-CI: 0.991–0.971) and 0.989 (95%-CI: 0.975–1.000), HC-ACT+ with AUROC of 0.935 (95%-CI: 0.884–0.987) and 0.937 (95%-CI: 0.889–0.984), HC-ACT-LR with AUROC of 0.892 (95%-CI: 0.836–0.948) and 0.880 (95%-CI: 0.819–0.941), and finally HC-aPTT with AUROC of 0.885 (95%-CI: 0.823–0.948) and 0.839 (95%-CI: 0.766–0.912). ROC curves of laboratory-based anti-Xa activity and HC-POCT-based coagulation assays are displayed in Fig. 2. Diagnostic accuracy at the ideal cut-off values is summarized in Table 2 and visualized in Fig. 3. Misprediction percentage for ruling out edoxaban plasma concentrations > 30 and > 50 ng/mL was 0% when using laboratory-based calibrated anti-Xa activity, but 1.4 and 3.2%/2.9 and 4.8%/2.3 and 4.8%/4.3 and 4.8%, respectively, when using HC-INR/HC-ACT+/HC-ACT-LR/HC-aPTT.

**Table 2:** Diagnostic accuracy of Hemochron™ Signature Elite POCT for edoxaban

Treatment threshold	Ideal cut-off	Specificity, %	Sensitivity, %	MP, %	LR	PPV, %	NPV, %
≤30 ng/mL	lab-basedcalibratedanti-Xa activity ≤ 30 ng/mL	100.0(91.7–100)	94.0(82.5–98.4)	0.0	–	100(90.6–100)	94.7(84.5–98.6)
	HC-INR ≤ 1.5	98.6(91.2–99.9)	98.0(88.0–99.9)	1.4	68.6(9.8–480.4)	98.0(88.0–99.9)	98.6(91.2–100)
	HC-ACT+ ≤ 115 s	97.1(89.1–99.5)	59.2(44.3–72.7)	2.9	20.7(5.2–82.8)	93.5(77.2–98.9)	77.3(66.9–85.2)
	HC-ACT-LR ≤ 105 s	97.1(89.1–99.5)	38.3(24.9–53.6)	2.3	13.4(3.3–55.1)	90.0(66.9–98.2)	70.1(59.8–78.8)
	HC-aPTT ≤ 68 s	95.7(87.0–98.9)	50.0(35.4–64.6)	4.3	11.5(3.7–36.0)	88.9(69.7–97.1)	73.3(62.8–81.9)
≤50 ng/mL	lab-basedcalibratedanti-Xa activity ≤ 50 ng/mL	100(90.6–100)	89.5(77.8–95.6)	0.0	–	100(91.3–100)	88.7(76.3–95.3)
	HC-INR ≤ 2.1	96.8(88.0–99.4)	96.5(86.8–99.4)	3.2	30.4(7.8–119.0)	96.5(86.8–99.4)	96.8(88.0–99.4)
	HC-ACT+ ≤ 120 s	95.2(85.8–98.8)	57.1(43.2–70.0)	4.8	12.0(3.9–37.1)	91.4(75.8–97.8)	71.4(60.4–80.5)
	HC-ACT-LR ≤ 110 s	95.2(85.8–98.8)	48.1(34.5–62.0)	4.8	10.1(3.2–31.6)	89.7(71.5–97.3)	68.2(57.3–77.5)
	HC-aPTT ≤ 68 s	95.2(85.8–98.8)	44.4(31.2–58.5)	4.8	9.3(3.0–29.3)	88.9(69.7–97.1)	66.7(55.9–76.0)

HC-INR, HC-ACT+, HC-ACT-LR, HC-aPTT = Hemochron™ Signature Elite point-of-care test system-based international normalized ratio, activated clotting time, and activated partial thromboplastin time; *MP* Misprediction percentage; *LR* Likelihood ratio; *PPV* Positive predictive value; *NPV* Negative predictive value. 95%-confidence intervals are reported in brackets wherever applicable

## Discussion

**Fig. 4:**
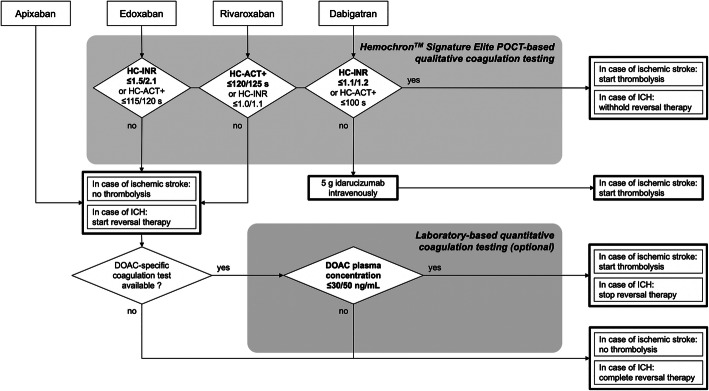
Proposed algorithm for emergency coagulation assessment using the Hemochron™ Signature Elite point-of-care test system (POCT) for rapid decision making in patients treated with direct oral anticoagulants (DOAC). Ideal assay- and DOAC-specific cut-offs are provided for the 30/50 ng/mL thresholds. ICH = intracranial hemorrhage; HC-INR and HC-ACT+ = Hemochron™ Signature Elite point-of-care test system-based international normalized ratio and activated clotting time

This study completes our evaluation of the Hemochron™ Signature Elite POCT for global coagulation testing in DOAC-treated patients (Ebner, Birschmann, Peter, Spencer, et al., 2017). We were able to demonstrate that HC-POCT results correlate with actual edoxaban plasma concentrations, and may be used to exclude even very low edoxaban concentrations in a real-life blood sample at the bedside with high specificity (> 95%) by establishing ‘ideal’ assay-specific cut-offs for the 30 and 50 ng/mL treatment thresholds. These ideal cut-offs may well be different to the assays’ reference ranges. Importantly, HC-INR’s diagnostic accuracy for ruling out elevated edoxaban concentrations in a blood sample was comparable to that of laboratory-based calibrated anti-Xa activity (see Table 2, and Figs. 2 and 3), which is mostly recommended for assessment of coagulation during edoxaban therapy [5, 6]. HC-INR and HC-ACT+ are of special interest, as they are both not only influenced by edoxaban but also by rivaroxaban and dabigatran (Ebner, Birschmann, Peter, Spencer, et al., 2017). Therefore, for the purpose of providing physicians a comprehensive guideline for emergency assessment of the coagulation status in DOAC-treated patients (see Fig. 4), we recalculated the diagnostic accuracy of HC-INR and HC-ACT+ for rivaroxaban at the currently recommended treatment thresholds (see Supplemental Table 1 using data collected during an earlier study [8, 9]. Whilst the ideal cut-off for HC-INR and rivaroxaban was only found at very low POCT results, where sensitivity is insufficient, HC-ACT+ performs well with a sensitivity of 67.4% and even 81.1% at the 30 and 50 ng/mL threshold, respectively (see Supplemental Table 1). This leads to the conclusion that rivaroxaban has a much weaker effect on HC-INR than edoxaban. Varying effects of the different factor Xa inhibitors to global coagulation assays [8–10] and also anti-Xa activity [19] are well noted in the literature, however, to the best of our knowledge, there has not been a conclusive explanation for the different reactivity. Unfortunately, apixaban is not covered by any HC-POCT-based coagulation assay [8, 9]. In synopsis, and in accordance with the results of our previous evaluation of the Hemochron™ Signature Elite POCT [8, 9], to speed up decision making in the emergency department, we propose a two-assay approach using HC-INR for ruling out elevated levels of dabigatran or edoxaban, and HC-ACT+ for the exclusion of rivaroxaban in a blood sample (see Fig. 4). At the dispense of a lower sensitivity, each of the two assays may also be used on its own for all three substances. It needs to be kept in mind, however, that different cut-offs will have to be applied for the 30 and 50 ng/mL (or any other future) threshold and for each DOAC.

### Strengths and limitations

All coagulation testing was conducted using real-life patient samples and edoxaban plasma concentrations around the 30 and 50 ng/mL treatment thresholds are well represented in the dataset, which supports the validity of the presented study. Only few data points were lost to analysis. The aim of the study was to evaluate the ability of HC-POCT-based coagulation assays to exclude clinically relevant edoxaban plasma concentrations in the emergency situation. The samples we analyzed were, however, taken sequentially from only 20 patients in a non-emergency setting. This was done for reasons of feasibility and in order to be able to obtain and analyze a wide range of low edoxaban plasma concentrations.

Sensitivity of HC-POCT is limited. Higher diagnostic accuracy may be achieved by using calibrated anti-Xa activity (see Figs. 1, 2, and 3, and Table 2). Relying solely on laboratory-based testing, however, may critically impede or even prevent thrombolysis if the time window is exceeded, and sole suspicion of DOAC intake may lead to unnecessary and potentially harmful reversal therapy in up to 27% of patients without relevant DOAC plasma levels at the time of emergency admission [20].

In this study we tested around two edoxaban thresholds, which have been endorsed by authors of clinical guidelines [5, 12, 13]. These thresholds are, however, still not supported by prospective clinical data.

The ideal assay-specific cut-offs suggested in this manuscript were established retrospectively and warrant prospective clinical evaluation. Also, they are not transferable to other prothrombin time/INR- or ACT-based POCT devices or laboratory-based assays, as different reagents are used [8–11].

It is important to note that in order to use HC-INR or HC-ACT+ to exclude relevant DOAC plasma concentrations, the type of DOAC and the approximate time of the last dose must be known. Otherwise, relevant DOAC concentrations (e.g., of apixaban [10]) may be overlooked or drug levels might still be on the rise during the first hours after intake.

## Conclusion

This study represents the first evaluation of coagulation testing in edoxaban-treated patients using the commercially available Hemochron™ Signature Elite POCT and completes our previous evaluation of this POCT regarding monitoring of DOAC [8, 9]. HC-ACT+ and most accurately HC-INR may be used to rule out even very low concentrations of edoxaban in a blood sample, potentially identifying patients who may be treated with thrombolysis in case of acute ischemic stroke or undergo urgent surgery, and patients with serious bleeding in whom administration of a (possibly prothrombotic) anticoagulation reversal agent might be avoided. Using a sufficiently low cut-off of ≤1.5 or ≤ 2.1 for the 30 and 50 ng/mL threshold, respectively, HC-INR is capable to identify these patients in 98 and 97% of cases whilst providing the necessary safety by reliably detecting elevated edoxaban plasma concentrations in > 95%. However, as the suggested cut-offs were determined retrospectively, further evaluation in a prospective clinical trial – ideally in the emergency situation – is warranted.

## Supplementary Information

**Additional file 1.**

## Data Availability

The datasets used and/or analyzed during the current study are available from the corresponding author on reasonable request.

## Abbreviations

ACT: Active clotting time
aPTT: Activated partial thromboplastin time
AUROC: Area under the receiver operating curve
HC-POCT: Hemochron™ signature elite point-of-care test system
INR: International normalized ratio
ROC: Receiver operating curve
