# Supplementary material for: Point-of-care testing for emergency assessment of coagulation in patients treated with direct oral anticoagulants including edoxaban
Source: Neurol Res Pract. 2021 Mar 1;3:9. doi: 10.1186/s42466-021-00105-4 (PMC7919064; doi:10.1186/s42466-021-00105-4)
Supplement: Supplementary file 1 — Additional file 1. [file 42466_2021_105_MOESM1_ESM.docx]

**Supplemental Table 1: Diagnostic accuracy of Hemochron^TM^ Signature Elite POCT for rivaroxaban**

| Treatment threshold | Ideal cut-off | Specificity, % | Sensitivity, % | MP, % | LR | PPV, % | NPV, % |
| --- | --- | --- | --- | --- | --- | --- | --- |
| ≤30 ng/mL | HC-INR ≤1.0 | 97.2 (89.3–99.5) | 32.6 (20.0–48.1) | 2.8 | 11.6 (2.8–48.3) | 88.2 (62.3–97.9) | 69.0 (58.9–77.7) |
|  | HC-ACT+ ≤120 s | 95.7 (87.2–98.9) | 67.4 (51.9–80.0) | 4.4 | 15.7 (5.1–48.4) | 91.2 (75.2–97.7) | 81.7 (71.3–89.1) |
| ≤50 ng/mL | HC-INR ≤1.1 | 95.2 (85.8–98.8) | 46.3 (32.8–60.3) | 4.8 | 9.7 (3.1–30.4) | 89.3 (70.6–97.2) | 67.4 (56.6–76.8) |
|  | HC-ACT+ ≤125 s | 96.8 (88.0–99.4) | 81.1 (67.6–90.1) | 3.2 | 25.6 (6.5–100.6) | 95.6 (83.6–99.2) | 85.9 (75.2–92.7) |

HC-INR and HC-ACT+ = Hemochron^TM^ Signature Elite point-of-care test system-based international normalized ratio and activated clotting time; MP = misprediction percentage; LR = likelihood ratio; PPV = positive predictive value; NPV = negative predictive value. 95%-confidence intervals are reported in brackets wherever applicable. The presented ideal cut-offs and the diagnostic accuracy parameters of both assays for rivaroxaban have been recalculated for the current 30 and 50 ng/mL treatment thresholds using data collected during an earlier study (Ebner et al., 2017).

**Supplemental Reference**

Ebner, M., Birschmann, I., Peter, A., Spencer, C., Hartig, F., Kuhn, J., Blumenstock, G., Zuern, C. S., Ziemann, U., & Poli, S. (2017, Feb 15). Point-of-care testing for emergency assessment of coagulation in patients treated with direct oral anticoagulants. *Crit Care, 21*(1), 32. <https://doi.org/10.1186/s13054-017-1619-z>
